# Natural Inhibitors of Snake Venom Metalloendopeptidases: History and Current Challenges

**DOI:** 10.3390/toxins8090250

**Published:** 2016-08-26

**Authors:** Viviane A. Bastos, Francisco Gomes-Neto, Jonas Perales, Ana Gisele C. Neves-Ferreira, Richard H. Valente

**Affiliations:** 1 Laboratory of Toxinology, Oswaldo Cruz Foundation (FIOCRUZ), Rio de Janeiro 21040-900, Brazil; vivika.bastos@gmail.com (V.A.B.); gomes.netof@gmail.com (F.G.-N.); jperales@ioc.fiocruz.br or jonasperales@gmail.com (J.P.); anag@ioc.fiocruz.br or anagextra@gmail.com (A.G.C.N.-F.); 2 National Institute of Science and Technology on Toxins (INCTTOX), CNPq, Brasilia 71605-001, Brazil

**Keywords:** cross-linking, hydrogen/deuterium exchange, mass spectrometry, metalloendopeptidase inhibitor, modeling, natural immunity, natural resistance, snake venom, structure, therapeutic application

## Abstract

The research on natural snake venom metalloendopeptidase inhibitors (SVMPIs) began in the 18th century with the pioneering work of Fontana on the resistance that vipers exhibited to their own venom. During the past 40 years, SVMPIs have been isolated mainly from the sera of resistant animals, and characterized to different extents. They are acidic oligomeric glycoproteins that remain biologically active over a wide range of pH and temperature values. Based on primary structure determination, mammalian plasmatic SVMPIs are classified as members of the immunoglobulin (Ig) supergene protein family, while the one isolated from muscle belongs to the ficolin/opsonin P35 family. On the other hand, SVMPIs from snake plasma have been placed in the cystatin superfamily. These natural antitoxins constitute the first line of defense against snake venoms, inhibiting the catalytic activities of snake venom metalloendopeptidases through the establishment of high-affinity, non-covalent interactions. This review presents a historical account of the field of natural resistance, summarizing its main discoveries and current challenges, which are mostly related to the limitations that preclude three-dimensional structural determinations of these inhibitors using “gold-standard” methods; perspectives on how to circumvent such limitations are presented. Potential applications of these SVMPIs in medicine are also highlighted.

## 1. Introduction

Snakes and their venoms have always driven the fascination and curiosity of mankind—including the desire to freely handle them without being harmed by the venomous effects of their bites. Members of some ancient tribes used to drink small amounts of venom seeking protection from future envenomation; curiously, individuals from other tribes were thought to be resistant as a consequence of having snake blood running through their veins [1].

The idea that snakes could be resistant to their own venom traces back to Greek philosophers and physicians. Galen of Pergamum (131–*ca*. 201 A.D.) described in his treatises *De antidotis* and *De Theriaca ad Pisonem* the recipe for a concoction named “Theriac of Andromachus”, which consists of a variety of ingredients including viper’s flesh. This theriac was believed to be, amongst other things, an antidote to snakebite [2,3]. Many centuries later, Felice Fontana (1730–1805), an abbot from Trentino (Italy) [4,5], inoculated the venom of the common European viper (*Vipera berus*) into the viper itself. He observed that the animal did not display any symptoms of envenomation, even after thirty six hours, leading to his celebrated aphorism “*Il veleno della Vipera non è veleno per la propria specie*” [6], later translated by Joseph Skinner to “The venom of the viper is not a poison to the viper itself” [7].

Based on the viper’s resistance to its own venom, Fontana concluded that this phenomenon was limited to the same species. Later, Guyon (1861) inoculated the venom of the viper into different snake species and found this resistance to be inter-specific [8]. By the end of the nineteenth century, Calmette, Phisalix and Bertrand first described the natural resistance displayed by some mammals, such as the mongoose and the hedgehog, towards snake bite envenomation [9,10]. Reports of natural protection against snake venom pathophysiological effects were also published in relation to several snakes, such as rattlesnakes (*Crotalus* sp.) [11], *Thamnophis s. sirtalis*, *Pituophis s. sayi*, *Natrix taxipilota*, *T. sirtalis infernalis*, *Heterodon contortrix* [12], *Sistrurus c. catenatus* [13], *Lampropeltis getulus floridana* [14], *Pseudoboa cloelia* [15], *Crotalus atrox* [16], *C. adamanteus* [17], and several mammals from the Didelphidae [18,19]. For a comprehensive review on the early days of the natural resistance field, the reader is referred to the work by Domont et al. [20].

After discovering the phenomenon of natural resistance, researchers in the field began to investigate its underlying mechanism of action. It is now currently accepted that this resistance can be conferred through two non-mutually exclusive mechanisms. In the first type, the resistant animal displays mutation(s) in the receptor(s) targeted by the snake’s toxin(s), which prevent(s) the deleterious effect(s). The second mechanism, on which this review will focus, involves the occurrence of serum proteins that neutralize the toxins by forming noncovalent complexes, rendering them unable to exert their pathophysiological effects [21]. These natural inhibitors are distributed in two major classes—the phospholipases A_2_ inhibitors (PLIs), which effectively inhibit the neuro- and myotoxic effects of snake venoms (for comprehensive reviews see [22,23,24]), and the SVMPIs, which can suppress the hemorrhagic symptoms commonly associated with Viperidae envenomation. In 2002, it was proposed that such inhibitors may be an important feature of the innate immune system of those venom-resistant animals due to their structural similarity to other proteins that exert relevant functions in immunity, and for acting as ready-made soluble acceptors in the serum, thus constituting the first line of defense against snake venom toxins [25].

**Figure 1:**
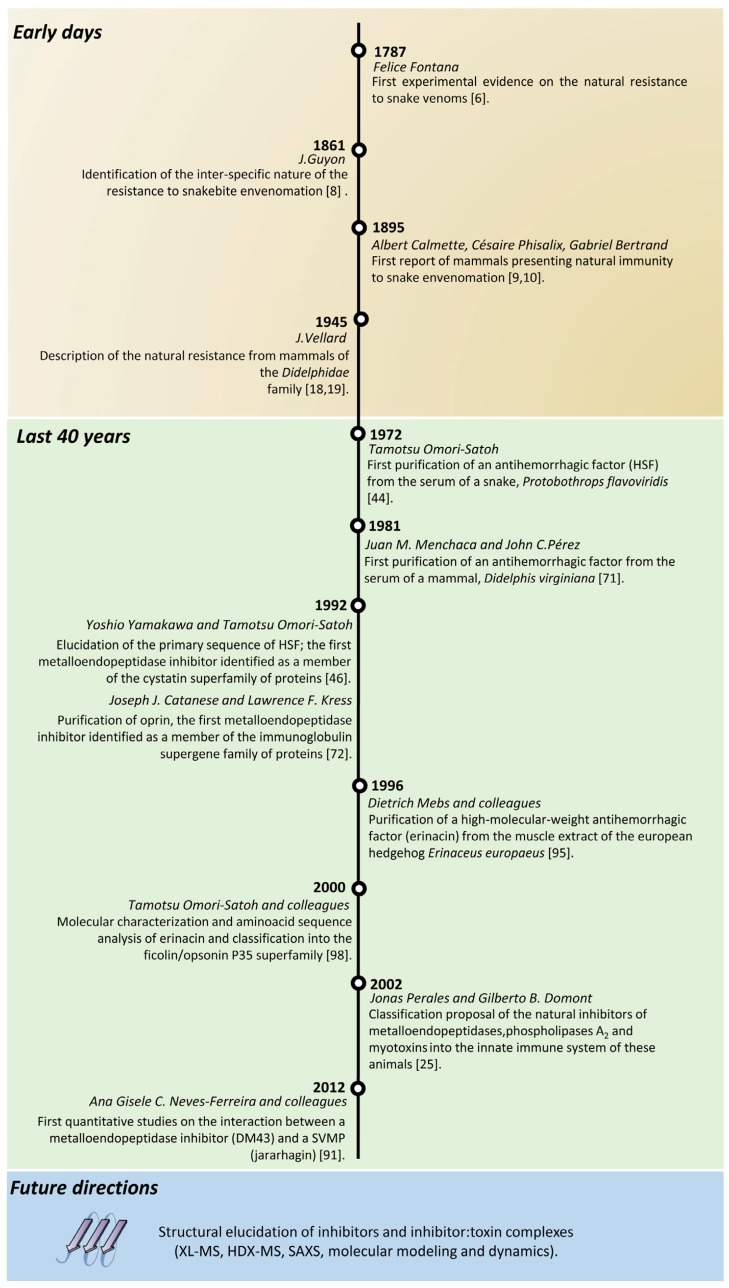
Research milestones on natural inhibitors of metalloendopeptidases. The investigation on the natural resistance that some animals presented to snake venoms began in the eighteenth century. Since Fontana’s pioneering work, the field has grown considerably. Researchers have managed to purify several inhibitors from the sera of snakes and mammals and determined their relevant physicochemical properties. The challenges that lie ahead are the three-dimensional structure elucidation of these snake venom metalloendopeptidase inhibitors (SVMPIs) in their free and toxin-complexed forms in order to better understand the molecular dynamics of this interaction.

**Figure 2:**
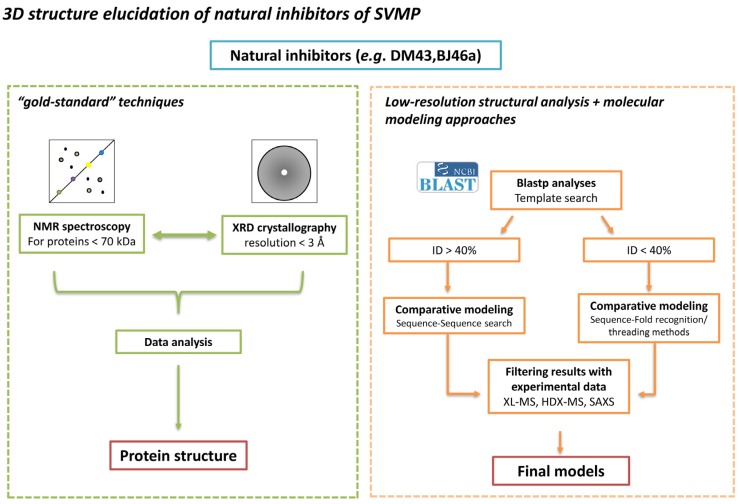
Strategies for a structural view of SVMPIs. (**Left**) The experimental methods for structure determination, NMR spectroscopy and XRD crystallography, are the “gold-standard” techniques in protein structure elucidation, providing atomic resolution of individual proteins and their complexes. The SVMPIs DM43 and BJ46a represent a challenge for these techniques. For NMR spectroscopy, due to the molecular size of both molecules, costly and time-consuming methods for sample labeling and analysis are required. For XRD crystallography, crystals of DM43 produced low-resolution diffraction pattern while BJ46a could not be crystallized, highlighting the limiting character of the crystallization step. Hence, modeling becomes an important tool for the structural studies of these molecules. (**Right**) In molecular modeling, the main step is the identification of a homologous protein, whose experimental structure has already been determined, to be used as a template structure. The identification in structure databases of sequences evolutionarily correlated with sequential identity greater than 40% is done by standard pairwise sequence search methods, allowing the generation of high accuracy models. However, below this sequence identity threshold the correlation between two structures is difficult to address. In this range, sequences are correlated directly with proteins of known structure (fold recognition). A drawback is that, due to the low evolutionary correlation and the low sensitivity in the sequence alignment building, the accuracy of the produced models is lower. On the other hand, the ensemble of models produced can be filtered according to their agreement with experimental data. In our proposed strategy, these data would come from XL-MS, HDX-MS and SAXS assays, leading to the selection of accurate models, and shedding some light on the three-dimensional structural characteristics of these SVMPIs. Consequently, the molecular basis of the interaction between the inhibitors and their target toxins could be established.

During the second half of the 20th century, a large portion of the research in this field has been devoted to the isolation of SVMPIs for further physicochemical and chemical characterizations, including primary structure determination. However, over the last 15 years, the main goal of natural resistance research shifted from protein purification to mechanistic studies in an attempt to understand the interaction between inhibitors and target toxins at the molecular level. This review does not intend to present all known SVMPIs and their determined characteristics; this information can be found by the reader in a historical series of reviews [20,21,24,26,27,28,29]. In fact, with this contribution, we aimed to summarize the available knowledge in the field of SVMPIs (Figure 1) and to discuss novel perspectives in this research area, especially on how to address the actual bottleneck due to the lack of information on the three-dimensional structures of SVMPIs (Figure 2).

## 2. Biochemical Background

### 2.1. Snake Venom Metalloendopeptidases (Metalloproteinases)

In the early days of experimental research on the effects of viperid envenomation, hemorrhage was recognized as one of its main clinical features [30]. At that time, the mechanism of hemorrhage was largely unknown, and some authors referred to the principle in snake venom that caused hemorrhage as “hemorrhagin” [31].

In 1960, Japanese investigators were able to purify peptidases from *Trimeresurus (Protobothrops) flavoviridis* venom that displayed hemorrhagic activity. Their functional assays showed that both the proteolytic and hemorrhagic activities of these proteins were eliminated following EDTA addition, indicating that these molecules were most likely metallopeptidases [32,33,34,35]. The atomic absorption spectroscopy experiments conducted by Bjarnason and Tu in 1978 confirmed this hypothesis, demonstrating that hemorrhagins are zinc-dependent metallopeptidases, containing 1 mol of zinc ion per mol of enzyme [36].

According to their structural features, snake venom metalloendopeptidases (SVMPs) are currently grouped into three main classes: PI, PII, and PIII. SVMPs belonging to the PI class present only the metalloendopeptidase domain in their structure, whereas the enzymes belonging to the PII class present an additional disintegrin domain. Members of the PIII class present metalloendopeptidase, disintegrin-like, and cysteine-rich domains; eventually, a lectin-like domain may be present (PIIId) [37].

Upon venom injection, SVMPs primarily target the capillary vessels, hydrolyzing components of the basement membrane and promoting apoptosis of endothelial cells, leading to the extravasation of blood components [38,39]. Together with other snake toxins, SVMPs can also promote dermonecrosis and inflammatory reactions [40]. The local effects prompted by SVMPs occur shortly after the bite and contribute prominently to the high morbidity rates observed in snakebite envenoming [41,42]. Apart from their contribution to the important tissue damage frequently observed at the site of venom injection, SVMPs may also trigger systemic effects, being key toxins to the pathophysiology of snake envenomation [43].

### 2.2. SVMPIs Isolated from Snakes

#### 2.2.1. Cystatin Superfamily (Fetuin-Like Proteins)

The first natural SVMPI purified from the sera of snakes was HSF (habu serum factor), from the serpent *Protobothrops (Trimeresurus) flavoviridis*. The purified protein has a molecular mass of 70 kDa, determined by ultracentrifugal sedimentation equilibrium, and an isoelectric point of 4.0. It inhibits the proteolytic activities of HR1 and HR2, two P-III class SVMPs isolated from the venom of this same snake, both in vivo and in vitro. Interestingly, no precipitin line was detected in immunodiffusion assays with the crude venom, HR1 or HR2, indicating that the neutralizing factor was not an immunoglobulin [44,45]. Indeed, HSF is a 323-amino acid-long glycoprotein that possesses two cystatin-like domains located in the *N*-terminal portion, followed by a *C*-terminal His-rich domain. MALDI-TOF MS (matrix-assisted laser desorption/ionization mass spectrometry) analysis of HSF showed that it has a molecular mass of 47,810 Da; compared to the native molecular mass (70 kDa), the results seem to indicate that HSF is homodimeric in solution [24,46,47]. Using molecular exclusion chromatography, Deshimaru and colleagues demonstrated that HSF binds the H6 protease from *Gloydius halys brevicaudus* venom at a 1:1 molar ratio; it also effectively inhibited several P-I, P-II and P-III class SVMPs from the venoms of *T. flavoviridis* (HR1A, HR1B, HR2a, HR2b, and H2) and *G. h. brevicaudus* (brevilysins H3, H4, H6, and L4), indicating that HSF has a broad inhibitory specificity, irrespective of the metalloendopeptidases’ domain architecture [48]. Recently, HSF was shown to interact with small serum proteins (SSP), i.e., low-molecular mass proteins from *T. flavoviridis* serum with unknown functions [49]. The interaction of HSF with SSP-1 allowed the inhibition of HV1, a P-III class SVMP isolated from the venom of this same snake. Neither HSF nor SSP-1 alone could inhibit HV1; it was only through the ternary complex among HSF, SSP-1 and HV1 that the toxin’s catalytic activity was abolished [50]. Currently, little is known about the regions of interaction between HSF and its target toxins. Aoki and co-workers have shown that the *N*-terminal half (residues 1–89) of the first cystatin-like domain of HSF is essential to its inhibitory activity. Additionally, molecular modeling analyses pinpointed a cluster of amino acid residues (Trp17, Trp48, Lys15, and Lys41) involved in the inhibition of SVMPs by HSF [51].

Another well-studied inhibitor from snake serum is BJ46a, isolated from *Bothrops jararaca*. BJ46a also presents two cystatin-like domains sharing 85% sequence identity with HSF. The glycoprotein’s primary structure, confirmed by both Edman degradation and cDNA sequencing, consisted of 322 amino acids with 12 cysteine residues and four *N*-glycosylation sites (Asn76, Asn185, Asn263, and Asn274). By MALDI-TOF MS, BJ46a showed a molecular mass of 46,101 Da; by molecular exclusion chromatography and dynamic laser light scattering, it has a calculated mass of 79 kDa, suggesting a homodimeric structure. BJ46a effectively inhibited the proteolytic activity of the P-III class SVMP jararhagin and the P-I class atrolysin-*C* upon the fluorogenic peptide Abz-Ala-Gly-Leu-Ala-Nbz; titration experiments using molecular exclusion chromatography indicated that the inhibitor interacted with those SVMPs at a 1:2 (BJ46a monomer:toxin) molar ratio. SDS-PAGE (sodium dodecyl sulfate polyacrylamide gel electrophoresis) analyses, under reducing and nonreducing conditions, showed a noncovalent interaction between the inhibitor and each target toxin. Interestingly, BJ46a was not able to interact with jararhagin-*C* [52], a processed form of the SVMP jararhagin possessing only the disintegrin-like and cysteine-rich domains, even at a three-fold molar excess of the inhibitor, suggesting that the toxin’s metalloendopeptidase domain is essential for BJ46a binding [53]. Upon complex formation, BJ46a dimer dissociated and each monomer noncovalently interacted with two molecules of metalloendopeptidase; thus, the inhibitor may have two toxin-binding sites for each monomer, a different stoichiometry than that reported for HSF [48]. A preliminary molecular modeling for BJ46a first cystatin domain was done, using HSF’s model as template. The results indicated that, in addition to the cluster of residues Trp17, Trp48, Lys15, and Lys41 (also found in the three-dimensional model of HSF), BJ46a has a second cluster formed by the residues Trp52 and Lys58 in the first cystatin-like domain that could be involved in the binding of a second toxin molecule; another possibility is the involvement of the second cystatin-like domain in the interaction, although these assumptions remain to be experimentally verified [28]. Recently, Shi and colleagues expressed BJ46a in the methylotrophic yeast *Pichia pastoris* [54]. This recombinant BJ46a (rBJ46a) showed a molecular mass of 58 kDa, although after treatment with endoglycosidase H (for the removal of high mannose glycans) its mass was reduced to 38 kDa (corresponding to the protein moiety). rBJ46a was able to reduce the invasion of B16F10 melanoma cells and MHCC97H hepatocellular carcinoma cells in an in vitro trans-well migration assay. In subsequent in vivo assays, rBJ46a partially inhibited tissue colonization in a lung cancer model (C57BL/6 mice infected with B16F10 cells) and reduced the occurrence of metastasis in BALB/c nude mice infected with MHCC97H cells. The authors attributed these antitumoral activities to rBJ46a inhibitory capacity towards matrix metalloendopeptidases (MMPs) 2 and 9, even though no clear evidence was presented to support this claim [55].

From the sera of the Chinese (*Gloydius blomhoffi brevicaudus*) and the Japanese (*G. blomhoffi*) mamushis, two inhibitors were purified: cMSF and jMSF. Both proteins have a molecular mass of 40,500 Da by MALDI-TOF MS and were also classified as members of the cystatin superfamily, presenting sequence identities of 84% (cMSF) and 83% (jMSF) when compared to HSF. However, these new inhibitors presented a 17-residue deletion within their *C*-terminal His-rich domain. Despite this deletion, the inhibitor cMSF suppressed mamushi venom-induced hemorrhage in a dose-dependent manner and inhibited the proteolytic activities of the P-III class SVMPs HR1A and HR1B from *Protobothrops flavoviridis* venom. As for jMSF, it interacted with brevilysins H2, H3, H4, and H6 from *G. blomhoffi brevicaudus* venom but was unable to inhibit the SVMP HR2a from *P. flavoviridis* venom and brevilysin L6 from *Agkistrodon halys brevicaudus*. Even though the previously mentioned *C*-terminal deletion did not affect cMSF anti-hemorrhagic activity, the authors demonstrated that cMSF has a lower thermal stability limit (60 °C) when compared to HSF (100 °C) [56]. Shioi and colleagues also managed to purify SSPs from the serum of the Japanese mamushi that, as above discussed for HSF, interacted with jMSF in a yet to be described mechanism [57]. 

From the sera of *P. flavoviridis* and *G. blomhoffii brevicaudus*, Aoki and coworkers purified and characterized three proteins: habu HLP from *P. flavoviridis* and HLP-A and HLP-B from *G. b. brevicaudus.* All three proteins showed sequence homology to HSF but were devoid of antihemorrhagic activity; therefore, those proteins were named HLP, standing for habu-like proteins. One of these HLPs (HLP-B) was able to inhibit calcium phosphate precipitation, characterizing it as a *bona fide* (snake) fetuin, a protein class that is known to interact with calcium and prevent calcification [58]. To map the protein regions responsible for the maintenance of the antihemorrhagic activity, the sequences of all SVMPIs belonging to the cystatin superfamily of proteins (BJ46a, HSF, cMSF, and jMSF) were aligned with the deduced HLP sequences. The first cystatin-like domain showed approximately 60% identity between inhibitors and HLPs, whereas the second domain was conserved amongst all proteins (84% to 94% identity), indicating that the diversification process that originated SVMPIs and HLPs resulted from an alteration in amino acid sequences in the first cystatin-like domain [59]. Finally, the authors propose that these three snake blood proteins from the fetuin family (SVMPIs, HLPs, and true fetuin) evolved via gene duplication from a common ancestor to achieve different functions, including conferring resistance against the deleterious effects of envenomation [59].

#### 2.2.2. Undetermined Protein Family

Additional inhibitors have been purified and partially characterized from the plasma/serum of venomous and non-venomous snakes, such as *Agkistrodon contortrix mokasen* [11,60], *Bothrops asper* [61], *Crotalus atrox* [62,63], *Dinodon semicarinatus* [64], *Natrix tesselata* [65], *Protobothrops mucrosquamatus* [66], and *Vipera palestinae* [67]. To date, none of them had their primary structure determined. However, the SVMPI isolated from *N. tesselata*, named NtAH, displayed unique structural characteristics. It is the only high-molecular-mass (880 kDa) metalloendopeptidase inhibitor isolated from snake blood displaying an oligomeric composition of three polypeptide chains of 150, 100, and 70 kDa in an unknown arrangement. NtAH inhibited BaH1, the main metalloendopeptidase from *Bothrops asper* venom [65].

### 2.3. SVMPIs Isolated from Mammals

The earliest reports of mammals with natural resistances to snake envenomation date back to the nineteenth century. In their experiments, Felix de Azara, Albert Calmette, Césaire Phisalix & Gabriel Bertrand described the immunities of the lutrine opossum (*Lutreolina*) [68], the mongoose (*Herpestes ichneumon*) [9], and the hedgehog (*Erinaceus europaeus*) [10], respectively.

Vellard [19], when studying the natural resistance that mammals of the family *Didelphidae* presented to snake venoms, proposed that such phenomenon should be an adaptation to prey on venomous snakes [69]. Based on his observations on the resistance of *Didelphis virginiana*, including the injection of a high dosage (15 mg/kg) of *Agkistrodon piscivorus* venom, Kilmon hypothesized that the only reason that this opossum could fight snakes and survive the venomous bites was the existence of a “unique and extremely efficient immune-response system” [70]. However, because there was no evidence of antibody involvement, the association with the immune system remained elusive.

In 1981, Menchaca and Pérez isolated an antihemorrhagic factor from *D. virginiana* serum, named AHF; this was the first antihemorrhagic factor to be purified from the serum of a mammal. AHF presented a molecular mass of 68 kDa, an isoelectric point of 4.1, thermal (0–37 °C) and pH (3–10) stabilities; no precipitin line formation was evident when AHF was incubated with rattlesnake venom, indicating that AHF did not interact with snake venoms in a classic antigen–antibody reaction [71].

#### 2.3.1. Immunoglobulin Supergene Family

In 1992, Catanese and Kress purified another inhibitor from *D. virginiana* serum, which was named oprin. It showed sequence homology (36% identity) with α_1_B-glycoprotein and was classified as a member of the immunoglobulin supergene family. Oprin was able to inhibit several snake venom metalloendopeptidases but failed to inhibit serine endopeptidases, MMPs or bacterial metalloendopeptidases; oprin interacted with *Crotalus atrox* α-proteinase. The authors proposed that oprin partially accounted for the natural resistance of *D. virginiana*, and that its serum would contain at least two inhibitors of metalloendopeptidases [72]. Other studies identified and characterized to different extents inhibitors belonging to the immunoglobulin supergene family from the plasma/serum of *Herpestes edwardsii* (AHF-1 to AHF-3) [73,74,75], *Lutreolina crassicaudata* [76], *Philander opossum* (PO41) [76,77], and *Didelphis albiventris* (DA2-II) [78].

Following the first studies on the resistance that *D. marsupialis* showed to snake venoms [79,80,81], Perales and colleagues optimized the purification of an antibothropic fraction (ABF) that effectively blocked the hemorrhagic and lethal effects of *Bothrops jararaca* venom in mice [82,83]. ABF was further fractionated yielding ABC (antibothropic complex), which was composed of two proteins, with apparent molecular masses of 48 kDa and 43 kDa, as determined by SDS-PAGE under reducing conditions. ABC inhibited the hemorrhagic, hyperalgesic and edematogenic effects of *Bothrops jararaca* venom [76,84,85]. In vitro, ABC inhibited the proteolytic activity of the venom upon fibrinogen, fibrin, collagen IV, laminin, and fibronectin [86]. ABC was also found in the opossum’s milk, reinforcing neonatal protection against snakebite envenomation [87].

Neves-Ferreira and colleagues fractionated ABC, leading to the isolation of two SVMPIs: DM40 and DM43. Both of these factors are acidic glycoproteins with molecular masses of 40,318 Da for DM40 and 42,373–43,010 Da for DM43 by MALDI-TOF MS; by SDS-PAGE under reducing conditions, DM40 and DM43 molecular masses are 43 kDa and 48 kDa, respectively [88]. DM43 remains the most extensively studied inhibitor to date; it is a homodimeric glycoprotein bearing three immunoglobulin-like domains per monomer and is homologous to α1B-glycoprotein, a human serum protein [89]. The structural resemblance and the presence of a degenerate WSXWS sequon on each domain of DM43 (typically found in proteins bearing an Ig-like fold) classified DM43 into the immunoglobulin supergene family of proteins [89]. The analysis of its glycan moiety revealed that all *N*-glycosylation consensus sites (Asn23, Asn156, Asn160, and Asn175) were occupied with complex-type *N*-glycans containing the monosaccharides *N*-acetylglucosamine, mannose, galactose, and *N*-acetylneuraminic acid at a 4:3:2:2 molar ratio, which is compatible with biantennary glycan chains.

MALDI-TOF MS analyses of deglycosylated and native DM43 revealed that the glycan moiety corresponded to 21% of the average molecular mass of the inhibitor [89,90]. Similar to many glycoproteins, DM43 presents at least four glycoforms, which may result from glycan composition heterogeneity [91]. In vitro, DM43 inhibited the proteolytic activity of the SVMP jararhagin upon the fluorogenic substrate Abz-Ala-Gly-Leu-Ala-Nba and upon casein, fibrinogen, and fibronectin; in vivo, DM43 showed the same properties as the ABC in mice [88]. Titration experiments using molecular exclusion chromatography and electrophoresis in denaturing conditions demonstrated that DM43 interacted with snake venom metalloendopeptidases at a 1:1 (monomer of DM43:toxin) molar ratio, and that this interaction was maintained noncovalently [88]. Surface plasmon resonance analysis using a sensor chip with immobilized jararhagin indicated a high-affinity interaction, with an equilibrium dissociation constant (*K*_D_) of 0.33 ± 0.06 nM [91]. The strength of the DM43-jararhagin binding was comparable to therapeutic monoclonal antibodies, which typically have *K*_D_ values in the range of 1 pM to 1 nM [92]. 

The current knowledge about the regions of interaction between DM43 and its target toxin is still very limited; DM43 does not bind jararhagin-*C*, indicating that the interaction between the inhibitor and target toxin involves the toxins’ metalloendopeptidase domain [89]. Additionally, after partial deglycosylation with PNGase F under nondenaturing conditions, the inhibitory activity of DM43 was reduced to 50% compared to native DM43 [90]. It still remains to be verified whether the *N*-glycans were directly involved with the interaction between the inhibitor and the metalloendopeptidase or if the partial removal of the *N*-glycosylation induced a conformational change that hindered the formation of the toxin–antitoxin complex.

The interaction between DM43 and SVMPs from different snake venoms was explored through affinity chromatography, with the covalent immobilization of DM43 on a HiTrap NHS-activated column. The venoms of *Bothrops atrox*, *B. jararaca*, *B. insularis*, and *Crotalus atrox* were injected into the DM43-column and the unbound and bound protein fractions were collected and analyzed through two-dimensional protein electrophoresis (2D-PAGE) [93]. DM43 was able to interact with several metalloendopeptidases from those venoms, but the presence of SVMP spots in the 2D-PAGE gels of the unbound fractions indicated that DM43 was not a universal SVMP inhibitor. Accordingly, DM43 did not interact with HF3, a highly glycosylated P-III class SVMP from *B. jararaca* venom, suggesting that some SVMPs may have structural features that pose difficulties to DM43 binding [94]. On the other hand, DM43 was able to interact with MMPs from osteoarthritis synovial liquid and supernatants of MCF-7 cell cultures; Western blot analyses have shown that DM43 interacted with MMP-2, MMP-3, and MMP-9, outlining a promising application for DM43 in biotechnology [95].

#### 2.3.2. Ficolin/Opsonin P35 Family

The antihemorrhagic factor erinacin was isolated from *Erinaceus europaeus* muscle extract. It is a high-molecular mass protein of 1040 kDa composed of two main subunits—α and β—at a molar ratio of 1α:2β. The α-subunit is a homodecamer of 370 kDa maintained by noncovalent bonds, and the β subunit is composed of ten polypeptide chains of 35 kDa interacting via covalent bonds [96]. When analyzed by electron microscopy, erinacin showed a molecular structure that resembled a flower bouquet, an arrangement typical of proteins from the ficolin/opsonin P35 superfamily, such as plasma ficolin and the Hakata antigen [97,98]. Amino acid sequencing revealed that both subunits of erinacin were composed of *N*-terminus, collagen- and fibrinogen-like domains homologous to proteins from this family [99]. In vitro, erinacin inhibited a metalloendopeptidase from the venom of *B. jararaca*, through the establishment of an equimolar complex; it did not inhibit serine endopeptidases such as trypsin or chymotrypsin, and the dissociation of erinacin into its subunits caused complete loss of its antihemorrhagic activity. Regarding the mechanism of metalloendopeptidase inhibition by erinacin, the authors suggested two possibilities: (a) the *C*-terminal region of the fibrinogen-like domain of erinacin could contribute to the metalloendopeptidase inhibition by recognizing an *N*-acetylglucosamine molecule, as reported for P35 lectin and plasma ficolin [97,100]; and (b) the collagen-like domain of erinacin would act as a “decoy” substrate for the SVMPs [99].

## 3. Possible Therapeutic Applications

SVMPs are members of the metzincin clan of metalloendopeptidases, together with ADAMs (a disintegrin and metalloendopeptidase), ADAMTS (ADAM with thrombospondin motifs), astacins, serralysins, and MMPs [101,102,103]. SVMPs are abundant toxins in Viperidae (and some Colubridae) venoms, being responsible for the onset of local (blistering, edema, inflammatory reactions, and dermonecrosis) and systemic (hemorrhage, coagulopathy, and myonecrosis) pathophysiological effects [43].

The current antiophidic therapy is based on intravenous administration of antivenom, which in turn relies on antibody specificity, affinity, and ability to reach SVMPs (and other snake venom toxins) to be effective. The application of antivenom soon after *B. jararaca* venom injection in mice was not able to fully reverse the local effects of envenomation due to impaired and delayed venom/antivenom interaction at the site of injury [104,105]. Therefore, one of the current initiatives for the improvement of antiophidic therapy is the local administration of inhibitors soon after the envenomation event to restrain the extent of tissue degradation, and thus lower the high morbidity rates associated with snakebite envenoming [106].

SVMPIs could be used as a scaffold for the rational development of peptidic inhibitors of metalloendopeptidases because the interaction between the inhibitors and their target toxins is specific and leads to a tight-binding complexation.

The concept of rational drug design has already been applied to proline-rich oligopeptides from *Bothrops jararaca* venom, known as bradykinin-potentiating peptides (BPPs), initially described by Ferreira and co-workers [107]. From this same venom, other authors were able to isolate and fully determine the primary structures of six BPPs [108], including <Glu-Trp-Pro-Arg-Pro-Gln-Ile-Pro-Pro, later named BPP-9a; a synthetic version of this peptide was named SC 20,881. Gavras et al. demonstrated that the parenteral administration of SC 20,881 to hypertensive patients led to a significant drop in arterial blood pressure [109]. However, due to its lack of oral activity, this peptide had limited clinical applicability [110]. In following studies, Ondetti and colleagues showed that BPPs were substrate analogs that bound competitively to the active site of angiotensin-converting enzyme (ACE), and the optimal inhibitory region of the sequence was composed by the tripeptide Phe-Ala-Pro [110]. Based on the Phe-Ala-Pro sequence and structural studies of carboxypeptidase A as a model for ACE, Cushman and Ondetti synthesized d-2-methylsuccinyl-l-proline. This molecule proved to be a specific inhibitor of ACE with an IC_50_ of 22 µM. A further substitution of a carboxyl to a sulfhydryl group enhanced the molecule’s inhibitory activity by three orders of magnitude, yielding the compound SC 14,225, later named Captopril [111,112]. Captopril is widely used in the treatment of hypertension and paved the way for the development of many antihypertensive compounds [113].

Peptide drugs are an ever-growing branch of the pharmaceutical industry, with a market value estimated at more than 40 billion dollars per year; these pharmaceuticals offer high potency, high selectivity, high chemical diversity, lower toxicity, and lower accumulation in tissues [114]. Peptide inhibitors of metalloendopeptidases are currently approved for the therapeutic intervention of hypertension, periodontal disease, and osteoarthritis [115]. Hence, peptide drugs derived from the natural inhibitors of metalloendopeptidases could not only be used in the improvement of the antiophidic therapy but also for the treatment of many pathological conditions related to the abnormal expression of closely related molecules, such as the ADAMs, ADAMTS and MMPs. These last are associated with the spread of malignant tumors and chronic diseases (e.g., multiple sclerosis, arthritis, fibrosis, and inflammatory conditions), whereas ADAMs/ADAMTS are involved in interstitial pulmonary fibrosis, bronchial asthma, and neurodegenerative diseases [115,116].

Most inhibitors of metalloendopeptidases undergoing clinical trials are small molecules that possess zinc-binding groups, such as hydroxamate, that interact with side pockets of the catalytic site; classical representatives of these low selectivity inhibitors are marimastat and batimastat. However, both peptidic inhibitors have been discontinued during phase III of clinical trials for the treatment of invasive cancers because they displayed an excessive number of off-target effects [115,117].

The research history on natural SVMPIs described in this review envisage the possibility that they possess a different mechanism of inhibition than the one described for the previously mentioned artificial inhibitors, targeting different regions of the molecule with higher specificity, as shown for some TIMPs (tissue inhibitors of metalloendopeptidases) [118]. This potentially opens a new path for the treatment of pathological conditions related to the unbalanced expression of metalloendopeptidases. However, the current lack of knowledge regarding the tertiary and quaternary structures of these natural inhibitors, as well as their regions of interaction with SVMPs, is the bottleneck in this research area, and precludes further understanding of their mechanism(s) of action.

## 4. Status Quo and Perspectives on Three-Dimensional Structure Determination for SVMPIs

In this section, we will discuss experimental and computational approaches that could be used to further the knowledge of the tridimensional structures of SVMPIs (Figure 2) and the mapping of the regions of interaction between these inhibitors and SVMPs.

To date, no three-dimensional structures have been experimentally determined for any members of the different protein families related to SVMPIs (cystatin, immunoglobulin supergene, and ficolin/opsonin P35). Furthermore, molecular modeling attempts have only been performed for one member of the immunoglobulin supergene family—DM43 [89]—and one from the cystatin superfamily—HSF [51]. Due to the lack of literature on the subject, the discussion that follows will rely on some of our group’s unpublished data, related to two well-characterized SVMPIs: BJ46a (isolated from *Bothrops jararaca*—cystatin superfamily) [53] and DM43 (from *Didelphis aurita*—immunoglobulin supergene family) [89].

During the first 10 years after the primary structures of BJ46a and DM43 were published, our efforts were focused on applying standard X-ray diffraction (XRD) protocols to study the crystallized forms of these proteins. Multiple attempts at crystallizing the inhibitors BJ46a and DM43 have failed; after the eventual successful crystallization (DM43 only), the crystals showed a low-resolution diffraction pattern. A possible explanation is that these SVMPIs are glycoproteins whose glycan antennae show high conformational heterogeneity. These different states can be co-crystallized and interfere destructively in the diffraction pattern, decreasing its final resolution. Additionally, the absence of homologous proteins with an already determined crystallographic structure makes it impossible to solve the structure by molecular replacement, requiring more time and investment in producing heterologous proteins labeled with heavy atoms to solve the phase problem [119].

We have also evaluated if these proteins were good candidates for analysis by nuclear magnetic resonance (NMR) spectroscopy. However, DM43 (43 kDa monomer) and BJ46a (46 kDa monomer) are homodimeric in solution, exhibiting molecular masses outside the limit of standard protocols for NMR, requiring triple isotopic labeling (^15^N, ^13^C, and ^2^H) in parallel with the selective/segmental labeling of specific amino acids, thus resulting in high production costs for NMR samples, and long data analysis times [120,121].

To overcome the absence of structural information, molecular modeling techniques can be used to produce models that could help us explain the mechanism of inhibition of SVMPIs. Molecular modeling is based on the assumption that proteins with similar primary sequences (defined by an identity threshold) should display matching three-dimensional structures and biological functions [122,123]. The limiting step in protein modeling is the identification of template sequences (homologous sequences) whose experimentally (XRD or NMR) determined structures are available. After the identification, the two sequences (target and template) are aligned. The coordinates and geometrical parameters of the template structure (in the aligned regions) are applied to the target sequence to generate the new model. Thus, the quality of the template/target alignment is essential to produce a biologically relevant model. Ideally, these two sequences must display a minimum of 40% sequence identity, with long aligned regions, and a low number of sequence alignment gaps [124].

The first application of modeling for SVMPI structure determination was done for DM43 [89]. This member of the Ig supergene family is composed of three Ig-like domains (D0, D1 and D2) for a total of 291 amino acid residues. At that time, the best template available was the inhibitory receptor (p58-cl42) for human natural killer cells, a two-domain protein whose Protein Data Bank identifier (PDB ID) is 1NKR. The overall sequence identity (taking only domains D1 and D2 into account) is 25.9% (Table 1). The low sequence identity level led to a difficult modeling process that required manual interference at all steps. The model allowed the prediction of the third domain (domain D2) as the one interacting with the metalloendopeptidases. However, the detailed SVMP interacting regions proposed by the model are not supported by low-resolution structural data (crosslinking resolved by mass spectrometry (XL-MS), hydrogen/deuterium exchange monitored by mass spectrometry (HDX-MS), and small angle X-ray scattering (SAXS)) recently acquired by our group.

Recent searches in the PDB database for structures analogous to DM43 now revealed PDB ID 5EIQ (Table 1), human OSCAR ligand-binding domain, as the best match. Released 17 years later than PDB ID 1NKR, the structure 5EIQ shows an increased identity level, and similar number of positive matches for the same DM43 region. Even though the expected value level (*E*-value) of alignment for DM43/5EIQ points to a good match (7 × 10^−18^), it is still limited to domains D1 and D2, in the same fashion as for the alignment DM43/1NKR (original model). Nevertheless, the alignment is still below the 40% sequential identity threshold, suggesting that no new structural information is present in the protein structure database that could suggest a new direction for DM43 molecular modeling.

We used the same analysis for the SVMPIs HSF and BJ46a, belonging to the cystatin superfamily and displaying 85% sequence identity between themselves. As can be seen from Table 1, the sequence pairwise search results yielded sequence identity levels below the homology-modeling threshold of 40%, a low number of aligned residues, and high e-values. Altogether, these results suggest that, for the time being, SVMPIs are still a challenge for the application of standard modeling techniques.

Another methodology for modeling was independently proposed with the seminal papers of Bowie, Jones, and Zhang [125,126,127]. This method is able to correlate two sequences that are evolutionarily distant (low sequence identity), based on the concept that the folding, and consequently the function, is more conserved than the primary structure. These authors introduced the term “threading”, a method where the target sequence is fitted onto the backbone coordinates of a known protein structure (the template). The fitting is scored by an energy potential, and the lowest energy fitting corresponds to the best model.

There is one report in the literature describing this structural modeling approach for HSF [51]. This paper used sequence-to-structure methods to thread the HSF sequence into template PDB ID 1G96. Applying the algorithm DELTA-BLAST [128], we were able to trace the new structures available since then (Table 2). As can be seen, since 2001 (release date of structure 1G96), three more structures that are structurally related to HSF were determined. All selected structures are members of the cystatin superfamily, in agreement with the prediction from HSF’s primary structure. Moreover, entry 4LZI is a convergent choice between several search algorithms (data not shown).

Finally, the same analysis was done for BJ46a (85% sequential identity with HSF), and, as expected, the results were very similar (Table 3). Three out of four structures selected as template to model HSF were also found in this case. However, even though the results are equivalent, there was a significant difference in the alignment with the secondary structure elements calculated using the threading algorithm (data not shown).

In summary, sequence-based methods (i.e., comparative homology modeling) can only produce good quality alignments and high accuracy models for closely related sequences (>40% identity). Below this identity level, threshold sequence-to-structure methods (i.e., fold recognition modeling or 3D-threading) show better performance. However, the low quality of the alignments may compromise the accuracy of the generated models. These limitations led to the development of hybrid strategies, which combine search algorithms based on sequence-profiling methods, and the energy potentials derived from threading methods. The new generation of fully automated servers for protein structure prediction is based on this hybrid strategy (genThreader, PSIPred, and i-Tasser), allowing the structure prediction of proteins at a proteome scale [129,130,131].

Hence, in order obtain confident structural models for these SVMPIs, we advocate that the overall strategy should be to apply sequence-to-structure methods to produce large ensembles of models, followed by validation against experimental data generated by XL-MS [132], HDX-MS [133], and SAXS [134,135,136] (Figure 2, Right panel).

## 5. Conclusions

The field of natural inhibitors of snake venom toxins has advanced considerably since the amazing phenomenon of innate venom resistance was first described more than two centuries ago. Currently, the physicochemical characteristics of the antiophidic proteins are known, but the molecular bases underlying their neutralizing properties are not quite well understood. For instance, translating this scientific knowledge into novel effective therapies (e.g., preventing snake envenomation morbidity) necessarily requires a deep understanding of the structure–function relationship. To tackle this challenge, greater emphasis should be placed on the concerted use of emerging structural biology techniques that are complementary to traditional approaches.

## Figures and Tables

**Table 1:** Template search for the SVMPIs DM43, HSF, and BJ46a. All structures identified as possible templates are below the threshold of 40% of sequence identity.

Feature	Ig Supergene Family		Cystatin Superfamily	
	DM43 (291)		HSF (323)	BJ46a (322)
Template (PDB ID)	1NKR	5EIQ	2KZX	2WBK
Release date	11 November 1998	25 November 2015	15 February 2012	4 April 2014
Number of aligned residues	193	192	98	55
*E*-value	4 × 10^−5^	7 × 10^−18^	1 × 10^−1^	5 × 10^−1^
Identity	(50/193) 26%	(71/192) 37%	(26/98) 27%	(15/55) 33%
Positives	(78/193) 40%	(91/192) 47%	(42/98) 42%	(29/55) 52%
Gaps	(15/193) 7%	(14/192) 7%	(5/98) 5%	(1/55) 1%
Aligned region	9–196	1–188	14–110	207–261

The complete primary structures of DM43, HSF, and BJ46a are composed of 291, 323, and 322 amino acids, respectively (numbers in parentheses). PSI-Blast search with default parameters (Expect threshold 10, Word size 3, Matrix BLOSUM62, Gap Costs Existence 11, Extension 1, PSI-BLAST threshold 0.005) were done against the PDB database. Template is the best hit, identified by its PDB ID number. Release date is the structure’s publication date in the database. *E*-value is the expected number of chances that the match is random. Three percentages are calculated relatively to the number of aligned residues: identity (exact match residues), positives (exact + homology match residues), and gaps (inserted spaces to allow the alignment). 1NKR: inhibitory receptor (p58-cl42) for human natural killer cells. 5EIQ: human OSCAR ligand-binding domain. 2KZX: A3DHT5 from *Clostridium thermocellum*, Northeast Structural Genomics Consortium Target CmR116. 2WBK: beta-mannosidase, Man2A.

**Table 2:** New HSF-correlated structures in the PDB database, using DELTA-Blast.

Feature	HSF (323)			
Template (PDB ID)	4LZI	3PS8	1R4C	1G96
Release date	26 February 2014	21 December 2011	21 September 2004	6 April 2001
Number of aligned residues	222	115	107	115
*E*-value	4 × 10^−55^	6 × 10^−35^	8 × 10^−36^	2 × 10^−34^
Identity	(31/222) 14%	(15/115) 13%	(17/107) 16%	(15/115) 13%
Positives	(67/222) 30%	(39/115) 33%	(32/107) 29%	(39/115) 33%
Gaps	(55/222) 24%	(8/115) 6%	(4/107) 3%	(8/115) 6%

DELTA-Blast search followed by PSI-Blast, with the default parameters described in Table 1. Template is the best hit, identified by its PDB ID number. Release date is the structure’s publication date in the database. *E*-value is the expected number of chances that the match is random. Three percentages are calculated relatively to the number of aligned residues: identity (exact match residues), positives (exact + homology match residues), and gaps (inserted spaces to allow the alignment). Despite the intermediate sequential identity value, the structure 4LZI shows the best sequence coverage (number of aligned residues) and positive matches, being the best template since structure 1G96. 4LZI: *Solanum tuberosum* multicystatin. 3PS8: L68V mutant of human cystatin C. 1R4C: *N*-truncated human cystatin C, dimeric form. 1G96: human cystatin C, dimeric form.

**Table 3:** BJ46a-correlated structures in PDB database, using DELTA-Blast.

Feature	BJ46a (322)		
Template (PDB ID)	4LZI	3PS8	1G96
Release date	26 February 2014	21 December 2011	6 April 2001
Number of aligned residues	226	115	115
*E*-value	3 × 10^−33^	3 × 10^−27^	6 × 10^−27^
Identity	(30/226) 13%	(14/115) 12%	(14/115) 12%
Positives	(65/226) 28%	(41/115) 35%	(41/115) 35%
Gaps	(55/226) 25%	(8/115) 6%	(8/115) 6%

DELTA-Blast search followed by PSI-Blast, with default parameters described in Table 1. Template |is the best hit, identified by its PDB ID number. Release date is the structure’s publication date |in the database. *E*-value is the expected number of chances that the match is random. Three percentages are calculated relatively to the number of aligned residues: identity (exact match residues), positives (exact + homology match residues), and gaps (inserted spaces to allow the alignment). Despite the intermediate sequential identity value, the structure 4LZI shows the best sequence coverage (number of aligned residues) and positive matches, being the best template since structure 1G96. 4LZI: *Solanum tuberosum* multicystatin. 3PS8: L68V mutant of human cystatin C. 1G96: human cystatin C dimeric form.

## Abbreviations

The following abbreviations are used in this manuscript:

ABC	AntiBothropic Complex
ABF	AntiBothropic Fraction
ADAM	A Disintegrin And Metalloendopeptidase
ADAMTS	ADAM with ThromboSpondin motifs
BPP	Bradikynin-Potentiating Peptide
HDX-MS	Hydrogen/Deuterium eXchange MS
HLP	Habu-Like Protein
HSF	Habu Serum Factor
Ig	Immunoglobulin
MALDI TOF	Matrix-Assisted Laser/Desorption Ionization
MS	Mass Spectrometry
MMP	Matrix MetalloendoPeptidase
PDB ID	Protein Data Bank IDentifier
PLI	PhosphoLipase A_2_ Inhibitor
NMR	Nuclear Magnetic Resonance
SAXS	Small-Angle X-ray Scattering
SDS-PAGE	Sodium Dodecyl Sulfate PolyAcrylamide Gel Electrophoresis
SSP	Small Serum Protein
SVMP	Snake Venom MetalloendoPeptidase
SVMPI	SVMP Inhibitor
TIMP	Tissue Inhibitor of MetalloendoPeptidase
2D-PAGE	Two-Dimensional PolyAcrilamide Gel Electrophoresis
XL-MS	Cross-Linking MS
XRD	X-ray Diffraction
